# Impact of a diet and activity health promotion intervention on regional patterns of DNA methylation

**DOI:** 10.1186/s13148-019-0707-0

**Published:** 2019-09-11

**Authors:** Elizabeth Hibler, Lei Huang, Jorge Andrade, Bonnie Spring

**Affiliations:** 1 Department of Preventive Medicine, Northwestern University Feinberg School of Medicine, 680 North Lakeshore Drive, Chicago, IL 60611 USA; 2 Center for Research Informatics, Biological Sciences Division, University of Chicago, 900 E. 57th. Street, Chicago, IL 60637 USA; 3 Department of Pediatrics, The University of Chicago, 900 E. 57th. Street, Chicago, IL 60637 USA

**Keywords:** Lifestyle, Randomized trial, DNA methylation, Diet, Physical activity

## Abstract

**Background:**

Studies demonstrate the impact of diet and physical activity on epigenetic biomarkers, specifically DNA methylation. However, no intervention studies have examined the combined impact of dietary and activity changes on the blood epigenome. The objective of this study was to examine the impact of the Make Better Choices 2 (MBC2) healthy diet and activity intervention on patterns of epigenome-wide DNA methylation. The MBC2 study was a 9-month randomized controlled trial among adults aged 18–65 with non-optimal levels of health behaviors. The study compared three 12-week interventions to (1) simultaneously increase exercise and fruit/vegetable intake, while decreasing sedentary leisure screen time; (2) sequentially increase fruit/vegetable intake and decrease leisure screen time first, then increase exercise; (3) increase sleep and decrease stress (control). We collected blood samples at baseline, 3 and 9 months, and measured DNA methylation using the Illumina EPIC (850 k) BeadChip. We examined region-based differential methylation patterns using linear regression models with the false discovery rate of 0.05. We also conducted pathway analysis using gene ontology (GO), KEGG, and IPA canonical pathway databases.

**Results:**

We found no differences between the MBC2 population (*n* = 340) and the subsample with DNA methylation measured (*n* = 68) on baseline characteristics or the impact of the intervention on behavior change. We identified no differentially methylated regions at baseline between the control versus intervention groups. At 3 versus 9 months, we identified 154 and 298 differentially methylated regions, respectively, between controls compared to pooled samples from sequential and simultaneous groups. In the GO database, we identified two gene ontology terms related to hemophilic cell adhesion and cell-cell adhesion. In IPA analysis, we found pathways related to carcinogenesis including PI3K/AKT, Wnt/β-catenin, sonic hedgehog, and p53 signaling. We observed an overlap between 3 and 9 months, including the GDP-l-fucose biosynthesis I, methylmalonyl metabolism, and estrogen-mediated cell cycle regulation pathways.

**Conclusions:**

The results demonstrate that the MBC2 diet and physical activity intervention impacts patterns of DNA methylation in gene regions related to cell cycle regulation and carcinogenesis. Future studies will examine DNA methylation as a biomarker to identify populations that may particularly benefit from incorporating health behavior change into plans for precision prevention.

**Electronic supplementary material:**

The online version of this article (10.1186/s13148-019-0707-0) contains supplementary material, which is available to authorized users.

## Background

Large epidemiologic studies demonstrate that lifestyle choices such as a healthy diet and recommended levels of physical activity are associated with reduced risk of many chronic diseases including cardiovascular disease, diabetes, and cancer [1–5]. However, we know less regarding what biological mechanisms drive these associations at the molecular level. Health behaviors have been hypothesized to impact disease risk through epigenetic mechanisms, such as DNA methylation [6–25]. However, no studies have examined the impact of a lifestyle intervention including both dietary and physical activity components on epigenome-wide patterns of DNA methylation in blood.

DNA methylation is the addition of a methyl group at cytosine-phosphate-guanine (CpG) genomic loci and can act to suppress subsequent gene expression [26–29]. Physiologic response to both dietary intake and physical activity includes epigenetic mechanisms such as DNA methylation [30–34]. Observational studies have reported associations between physical activity and variation in patterns of DNA methylation that may impact immune function and risk of disease [6–10, 12, 35, 36]. Moreover, physical activity interventions demonstrate that physiologic effects may include reduced DNA methylation of genes associated with biological mechanisms such as inflammation, oxidative stress, and immune function [15, 17–20, 25]. However, these physical activity and exercise studies did not account for the impact of diet on DNA methylation.

Dietary intake and dietary patterns are also associated with DNA methylation in observational and randomized intervention studies [11, 14, 37–39]. Studies demonstrate that high-quality dietary intake as well as low adherence to specific dietary patterns, such as the Mediterranean Diet, are associated with hypomethylation using measures of LINE-1, or global methylation [33, 34, 37]. Global hypomehtylation in blood is associated with overall genomic instability and risk of certain cancers [33, 34, 37]. However, the majority of physical activity or dietary studies either used a candidate gene approach to examine DNA methylation in a specific gene(s) or examined levels of global DNA methylation. The current study is the first to assess the combined impact of diet and activity intervention on epigenome-wide DNA methylation.

The objective of this study was to examine the impact of the Make Better Choices 2 (MBC2) intervention on patterns of epigenome-wide DNA methylation. This approach is novel because the majority of previous studies examined either physical activity or dietary intervention alone; none included a lifestyle intervention that combined exercise, sedentary time, and dietary intake. Moreover, few studies have examined the impact of lifestyle interventions on patterns of DNA methylation in the blood as a potential marker of impact on immune function. In contrast, most studies to date examined either adipose or skeletal muscle as the target tissue of interest. The MBC2 study offers the opportunity to identify novel DNA methylation patterns associated with participation in a health behavior change intervention across multiple time points. This study is the first to examine the impact of the MBC2 intervention, a combined physical activity and dietary intervention,on the blood epigenome.

## Results

As shown in Table 1, we found no statistically significant differences between the MBC2 population and the subsample with DNA methylation measured (*n* = 68) on baseline characteristics or the impact of the intervention on behavior change. The mean age of participants in the current study was ~ 40 years of age in both intervention groups and the control group. Overall, MBC2 participants were primarily women, and there was a greater frequency of women in the sample for DNA methylation compared to the overall study. However, this difference was not statistically significant. Both samples included primarily non-Hispanic/Latino individuals, but nearly equal frequencies of White vs. Black race.

**Table 1:** Baseline characteristics of the DNA methylation subsample from the MBC2 population

Participant characteristics	DNA methylation (*n* = 68)			No DNA methylation (*n* = 144)		
	Control (*n* = 12)	Simultaneous (*n* = 25)	Sequential (*n* = 31)	Control (*n* = 32)	Simultaneous (*n* = 59)	Sequential (*n* = 53)
Age, mean (SD)	41.9 (12.6)	41.4 (12.3)	41.6 (10.1)	40.2 (12.6)	40.5 (11.7)	40.5 (11.4)
BMI, mean (SD)	32.6 (6.9)	34.5 (8.0)	37.3 (7.5)	34.7 (8.5)	34.1 (9.2)	35.5 (11.0)
Female, *n* (%)	27 (87.1)	55 (80.9)	8 (66.7)	38 (71.7)	107 (74.3)	25 (78.1)
Ethnicity, *n* (%)						
Hispanic or Latino	4 (13.3)	6 (9.5)	1 (9.1)	5 (9.6)	14 (10.0)	5 (15.6)
Non-Hispanic or Latino	26 (86.7)	58 (92.1)	10 (90.1)	47 (90.4)	126 (90.0)	27 (84.4)
Race, *n* (%)						
White	16 (51.6)	30 (44.1)	5 (41.7)	24 (45.3)	57 (39.6)	14 (43.8)
Black	14 (45.2)	34 (50.0)	6 (50.0)	24 (45.3)	65 (45.1)	13 (40.6)
Asian	0	2 (3.1)	1 (8.3)	1 (1.9)	6 (4.2)	1 (3.1)
Other or multiple	1 (3.2)	2 (3.1)	0	4 (7.5)	16 (11.1)	4 (12.5)

In the parent study, both the sequential and simultaneous groups showed comparably large, sustained diet and activity improvements, as described previously [40]. Therefore, we compared the control group to pooled samples from the sequential and simultaneous groups. We identified no differentially methylated regions at baseline between the control versus intervention groups (data not shown). We present the top 10 differentially methylated regions along with any genes associated with that region, and gene function, at 3 and 9 months in Tables 2 and 3, respectively. At 3 versus 9 months, we identified 154 and 298 differentially methylated regions, respectively, between controls compared to pooled samples from sequential and simultaneous groups. At 3 months, 98 of the 154 (63.6%) regions showed a decrease in methylation versus 188 of the 298 (63.1%) regions at 9 months. We observed primarily different regions of significance on different chromosomes between time points. However, one region on chromosome 4 (position: 185369135–185370076) showed a significant decrease in methylation between the control and intervention groups at both time points (FDR < 0.01). This included three CpGs at both time points and the region maps to the *IRF2* gene. We also identified two regions within chromosome 1 (position: 228756711–228756714) that correspond to the *DUSP5P1* gene. This region showed significantly reduced methylation between the baseline and the 3-month time points. There were three regions at 3 months and two at 9 months with either unknown function or in open reading frame uncharacterized protein regions, respectively. We present the additional top 40 differentially methylated regions at 3 and 9 months in Additional file 1: Table S1 and S2, respectively.

**Table 2.** Top 10 differentially methylation regions for 3-month control vs. pooled sequential/simultaneous

Region coordinates	Genes in region	Gene function [42]	# CpGs	FDR	Stouffer	*β* (max)	*β* (mean)
chr1:228756711–228756714	*DUSP5P1*	Pseudogene for MAPK phosphatase, proposed as a tumor suppressor in hematopoietic cancers [45, 46].	2	0.0036	0.7466	− 0.1183	− 0.1183
chr19:37463254–37463280	*ZNF568*	Role in transcriptional repression during embryonic development. Potential role in sessile serrated colon polyps [48].	2	0.0013	0.7671	0.0787	0.0549
chr20:58662458–58662959	*Unknown*	N/A	2	0.0029	0.7729	− 0.0878	− 0.0791
chr3:119499749–119500059	*NR1I2*	Transcription factor regulating detoxification and xenobiotic metabolism [65].	2	0.0004	0.8361	0.0982	0.0625
chr1:229764652–229764706	*URB2*	Ribosomal biosynthesis	2	0.0059	0.8873	0.0622	0.0351
chr2:105990524–105991411	*FHL2*	Assembly of extracellular membranes and used in models estimating epigenetic aging [66–68].	3	0.0013	0.8891	0.0539	0.0448
chr5:488398–488527	*SLC4A4*	Sodium biocarbonate cotransporter. Regulation of intracellular pH and renal tubular function [69].	2	0.0141	0.8900	0.0660	0.0493
chr3:46599958–46600244	*Unknown*	N/A	2	0.0147	0.8921	0.0738	0.0568
chr1:187445635–187445942	*LINC01037*	Long intergenic non-protein coding RNA 1037	2	0.0233	0.8955	− 0.0617	− 0.0354
chr4:185369135–185370076	*IRF2*	Competitively inhibits the IRF1-mediated transcriptional activation of interferons alpha and beta. Decreased expression is associated with angiogenesis and inflammation [43, 44].	3	0.0029	0.8977	− 0.1266	− 0.1005

**Table 3:** Top 10 differentially methylation regions for 9-month control vs. pooled sequential/simultaneous

Region coordinates	Genes in region	Gene function [42]	# CpGs	FDR	Stouffer	*β* (max)	*β* (mean)
chr8:51306070–51306693	*SNTG1*	Mediates dystrophin binding. Associated with idiopathic scoliosis [49].	3	2.49E− 09	0.0904	0.2066	0.1012
chr3:150265617–150265620	*SERP1*	Protein folding and metabolism. Inhibition can downregulate the Wnt/β-catenin pathway in keratinocytes [70].	2	4.96E− 06	0.1754	0.0351	0.0327
chr3:44636619–44636844	*ZNF197*	Transcription factor with potential role in skeletal muscle energy metabolism following exercise [71].	2	0.0008	0.3556	0.0465	0.0267
chr22:49875144–49875295	*C22orf34*	Chromosome 22 Open Reading Frame 34, uncharacterized protein.	2	0.0040	0.3980	− 0.0775	− 0.0575
chr16:1477870–1478718	*C16orf91*	Chromosome 16 Open Reading Frame 91, uncharacterized protein.	3	0.0001	0.4290	− 0.0667	− 0.0434
chr19:7813422–7813963	*EXOSC3P2*	Pseudogene, unknown function.	3	3.81E− 06	0.4426	− 0.0800	− 0.0468
chr13:101173617–101174420	*PCCA*	Subunit of mitochondrial enzyme critical to energy metabolism [72].	8	4.01E− 11	0.4572	− 0.0929	− 0.0537
chr11:1903176–1903333	*LSP1*	Expressed in immune system cells and may regulate motility and transendothelial migration. Potential marker of hepatocellular carcinoma risk [73].	2	0.0047	0.4611	− 0.0586	− 0.0486
chr4:185369135–185370076	*IRF2*	Competitively inhibits the IRF1-mediated transcriptional activation of interferons alpha and beta. Decreased expression associated with angiogenesis and inflammation [43, 44].	3	0.0030	0.4752	− 0.1271	− 0.0100
chr4:175241684–175241753	*CEP44*	Centrosomal protein	2	0.0071	0.4980	− 0.1368	− 0.0847
chr17:72915897–72916509	*USH1G*	Auditory and visual function development [50].	3	0.0009	0.5005	− 0.0636	− 0.0595

We present the results from the pathway analysis in Tables 4 and 5. The analysis in the GO database (Table 4) identified two pathways related to hemophilic cell adhesion and cell-cell adhesion via the plasma membrane. The pathway included the following genes: *DAB1*, *NECTIN4*, *CDH4*, *ROBO1*, *CDH9*, *PCDHGA1-12*, and *PCDHGB1-2,4-6*. These genes primarily showed decreased methylation, but *DAB1*, *NECTIN4*, and *CDH9* demonstrated increased methylation. We did not identify any significant pathways in the KEGG database (data not shown). In the top 20 IPA canonical pathways (presented by timepoint in Table 5), we identified pathways related to immune function and carcinogenesis in various tissues including the inflammasome, PI3K/AKT signaling, Wnt/β-catenin, sonic hedgehog, and p53. We also observed an overlap in pathways between 3 and 9 months. This included differential methylation of regions related to GDP-l-fucose biosynthesis, 2-oxobutanoate degradation, estrogen-mediated cell cycle regulation, and PI3K/AKT signaling. Finally, variation between the time points also identified potential target pathways including those related to the bladder, ovarian, and colorectal cancer, specifically.

**Table 4:** Gene ontology results by time point from control versus pooled sequential/simultaneous

Time point	ID	Description	Gene ratio	*p* value	*P* adjusted
3 months	GO:0007156	Homophilic cell adhesion via plasma-membrane adhesion molecules	23/123	6.59E−25	1.28E−21
	GO:0098742	Cell-cell adhesion via plasma-membrane adhesion molecules	23/123	5.15E−21	4.95E−18
9 months	GO:0007156	Homophilic cell adhesion via plasma-membrane adhesion molecules	24/221	3.91E−20	1.23E−16
	GO:0098742	Cell-cell adhesion via plasma-membrane adhesion molecules	25/221	2.87E−17	4.50E−14

**Table 5:** Top 20 IPA Canonical Pathways by time point for control versus pool sequential/simultaneous

Ingenuity canonical pathways	−log (*p* value)	Ratio
3 months		
GDP-l-fucose Biosynthesis I (from GDP-d-mannose)	1.89	0.500
Methylmalonyl pathway	1.59	0.250
2-Oxobutanoate degradation I	1.49	0.200
PI3K/AKT signaling	1.33	0.024
GM-CSF signaling	1.08	0.027
Colanic acid building blocks biosynthesis	1.06	0.071
Wnt/β-catenin signaling	1.01	0.018
Mitochondrial l-carnitine shuttle pathway	0.98	0.059
Bladder cancer signaling	0.96	0.023
GADD45 signaling	0.93	0.053
Acute myeloid leukemia signaling	0.92	0.022
Inflammasome pathway	0.91	0.050
Pyrimidine deoxyribonucleotides de novo biosynthesis I	0.85	0.044
Estrogen-mediated S-phase entry	0.81	0.039
Sonic hedgehog signaling	0.76	0.035
Sperm motility	0.73	0.017
LXR/RXR activation	0.73	0.017
FXR/RXR activation	0.71	0.016
Circadian rhythm signaling	0.70	0.029
9 months		
GDP-l-fucose biosynthesis I (from GDP-d-mannose)	1.65	0.500
Estrogen-mediated S-phase entry	1.47	0.077
Glioblastoma multiforme signaling	1.45	0.031
p53 signaling	1.44	0.036
Methylmalonyl pathway	1.36	0.250
PI3K/AKT signaling	1.30	0.033
2-Oxobutanoate degradation I	1.26	0.200
Cell cycle regulation by BTG family proteins	1.19	0.054
NAD biosynthesis from 2-amino-3-carboxymuconate semialdehyde	1.18	0.167
nNOS signaling in skeletal muscle cells	1.13	0.050
Bladder cancer signaling	1.13	0.035
Phosphatidylcholine biosynthesis I	1.12	0.143
tRNA splicing	1.11	0.049
Ovarian cancer signaling	1.11	0.028
Role of p14/p19ARF in tumor suppression	1.09	0.048
Phosphatidylethanolamine biosynthesis II	1.06	0.125
Gustation pathway	1.05	0.027
GABA receptor signaling	1.05	0.032
Role of Oct4 in mammalian embryonic stem cell pluripotency	1.04	0.044

## Discussion

In this study, we examined the impact of the Make Better Choices 2 intervention promoting healthy changes in diet and physical activity on regional patterns of epigenome-wide DNA methylation. The results of this study demonstrate that 12 weeks of improved lifestyle choices, focused on dietary and physical activity changes, was associated with change in DNA methylation at regions of DNA with gene functions related to immune cell metabolism, tumor suppression, and overall aging. Moreover, improved health behaviors were associated with immune cell adhesion pathways as well as several pathways critical to normal cell function and carcinogenesis. Finally, we observed some consistencies with patterns of DNA methylation over time at 3 and 9 months, but we also observed critical differences that warrant further investigation.

Previous studies examined the impact of physical activity or dietary interventions on DNA methylation in blood or circulating blood lymphocytes, but few examined an intervention incorporating both health behaviors [6–10, 41]. For example, Nakajima et al. demonstrated that moderate-intensity walking for ≥ 26 min/day, 2 days/week among 436 healthy adults was associated with *ASC* methylation [35], which controls IL-1β and IL-18 inflammatory cytokine expression [36]. Similarly, in the Cardiovascular Health Study, higher physical activity was associated with an anti-inflammatory phenotype via *TNF* hypermethylation and *IL-10* hypomethylation [12]. Moreover, among adult endurance athletes and sedentary controls (*n* = 24), Liu et al. found 72 differentially expressed genes in blood leukocytes with genes in inflammation and oxidative stress pathways [10]. Chilton et al. found that treadmill running for 30 min was associated with higher *TERT* and *SIRT6* gene expression in leukocytes, which regulate telomeres [8]. In contrast, Morabia et al. found no difference in *IL-6* methylation in blood leukocytes between sedentary car commuting versus public transportation (*n* = 180 adults) [41]. These studies demonstrate the impact of physical activity and sedentary behavior on DNA methylation as a blood-based biomarker of molecular mechanisms.

Studies have also examined the impact of dietary interventions among healthy adults on DNA methylation in blood, but the majority to date has focused on LINE-1 or candidate gene measures of DNA methylation. Studies by Agodi et al. foundation that women (*n* = 177) aged 13–50 years with low fruit consumption or folate deficiency were more likely to have LINE-1 hypomethylation in blood leukocytes (OR 3.7, 95% 1.4–9.5 and OR 3.6, 95% CI 1.1–12.1, respectively) [33]. Moreover, among healthy women aged 15–80 years (*n* = 299), Barchitta et al. reported that high adherence to the Mediterranean Diet was associated with increase in LINE-1 DNA methylation in whole blood (*β* = 0.573, *p* < 0.001) [34]. A recent systematic review by Aronica et al. also reported 12 intervention studies that examined the impact of dietary intervention studies on DNA methylation, but all of these examined methylation of candidate gene only [25]. Overall, these studies report that adherence to a Mediterranean Diet and/or calorie restriction were associated with decreased DNA methylation in obesity-related genes such as *PDK4*, *KCNQ1*, *WT1*, *SH2B1*, *FTO*, *BDNF*, and *PPARGC1A* or inflammatory genes such as *TNF-α*. However, none of the studies assessed epigenome-wide patterns of DNA methylation or dietary interventions that incorporated a physical activity component.

Although some prior research examined the effect of “lifestyle” interventions on DNA methylation, only a few of these interventions included both dietary and activity components and measured methylation from blood samples. For example, Molares et al. conducted a 10-week exercise and dietary intervention among 24 adolescents and examined patterns of whole blood DNA methylation from the Infinium Human Methylation 27 k BeadChip. The results showed that high response, as defined by reduction in BMI, was associated with differential methylation of individual CpG probes in the *AQP9*, *DUSP22*, *HIPK3*, *TNNT1*, and *TNNI3* genes (*p* < 0.05). These results did not, however, incorporate adjustment for multiple comparisons. Another study by McEwen et al. examined DNA methylation (Illumina 450 k) in peripheral blood mononuclear cells (PBMCs) among postmenopausal women aged 55–70 following a 6-month “lifestyle” intervention that included nine 2-h sessions focusing on health education and physical activity to reduce sedentary behavior [17]. The control group participated in six 1-h sessions that focused on education related to other topics [17]. The results showed no significant associations between DNA methylation and physical activity. Another study by Delago-Cruzata et al. examined the impact of a 6-month physical activity and dietary intervention on LINE-1 or global DNA methylation in PBMCs among a diverse population of sedentary, breast cancer survivors [16]. The intervention focused on increasing physical activity to 90 min/week, reducing caloric intake to 1200 kcal/day, and altering the nutrient distribution of the diet to 45% protein/30% carbohydrate/25% fat. The study found that LINE-1 DNA methylation was statistically significantly elevated at 6 and 12 months compared to baseline (75.5% vs. 78.5% and 77.7%, respectively; *p* < 0.001). Generalized estimated equation models also demonstrated that increased fruit/vegetable intake was associated with an increase in DNA methylation (*β* = 0.0118, 95% CI = 0.002–0.022) [16]. As described, few studies have examined epigenome-wide methylation in blood, and none to date have used the EPIC 850 k chip. Moreover, the majority of dietary interventions have tested caloric restriction to produce weight loss as opposed to improved dietary quality, such as increased consumption of fruits and vegetables, and the structure of physical activity interventions also varied. Nonetheless, the results of previous studies are consistent with the present findings in suggesting that lifestyle interventions designed to improve diet and physical activity can change patterns of DNA methylation in blood cells.

We identified over 150 differentially methylated regions at both time points. Within the top 10 most significant regions, the only consistent finding was that methylation of the region on chromosome 4 (position: 185369135–185370076) (FDR = 0.003) decreased at both time points (*β*_max_ = − 0.1266 and − 0.1271 at 3 and 9 months, respectively). This region includes the *interferon regulatory factor 2* (*IRF2*) gene, which inhibits IRF1-mediated transcriptional activation of interferons alpha and beta [42]. We observed a decrease in methylation following the MBC2 intervention, which may be associated with increased expression of IRF2 and, in turn, decreased angiogenesis and inflammation [43, 44].

The other top regions varied between the 3- and 9-month time points, but include genes with a known function related to overall health and disease risk. At 3 months, we identified two regions on chromosome 1 associated with decreased methylation of the *DUSP5P1* pseudogene in the family of MAPK phosphatases. Studies suggest that this gene may act as a tumor suppressor in hematopoietic malignancies [45, 46]. In the current study, we observed a decrease in methylation following the MBC2 intervention, which may indicate an upregulation of DUSP5P1 and tumor suppression. Kong et al. found that, among non-small cell lung cancer cases (*n* = 37) compared to controls (*n* = 17), *NR1I2* (aka *PXR*) expression was significantly higher among non-small cell lung cancer tumors compared to peripheral blood mononuclear cells [47]. We also observed that methylation increased in this region following MBC2, which may indicate that a healthier lifestyle downregulates the protein in immune cells. In contrast, a small study by Andrew et al. found that *ZNF568*, a zinc finger transcription factor, was hypermethylated in sessile serrated colon polyps (*n* = 34) compared to normal adjacent mucosal tissue (*n* = 15) [48]. We also observed an increase in *ZNF568* methylation following improved health behaviors at only 9 months. However, gene function may be tissue-specific and we identified no studies that examined DNA methylation of many of the top 10 genes in cells from whole blood.

At the 9-month time point, we also observed differentially methylated gene regions. The *SNTG1* gene is a cytoplasmic membrane protein that associates with the Duchenne muscular dystrophy gene, dystrophin [42]. Studies have shown that mutations leading to reduced production of this protein are associated with idiopathic scoliosis [49]. We observed an increase in methylation of *SNTG1* at 9 months, which could lead to decreased expression of the protein. We also observed decreased methylation of the *USH1G*, a gene critical to proper visual and auditory function [50]. Moreover, we observed increased methylation of the *PCCA*, which is an enzyme critical to proper function of the tricarboxylic acid (aka Krebs or TCA) cycle and energy production in muscle, both cardiac and skeletal [51, 52]. In circulating blood leukocytes, the TCA cycle is upregulated in the presence of proinflammatory signals [53]. However, studies also show that habitual healthy lifestyle reduces methylation of proinflammatory genes [12]. Thus, the observed increase in *PCCA* methylation may reflect a decrease in systemic inflammation following MBC2 participation, which should be measured in future studies. Overall, the present results are consistent with other studies suggesting that the types of diet and activity improvements produced in the MBC2 study affect molecular processes that play an integral role in promoting overall health.

Pathway analysis also identified gene pathways that may be important for understanding the impact of lifestyle on biological mechanisms that affect risk for developing chronic diseases. We identified the cell adhesion pathway in the GO database as significant at both 3 and 9 months. The importance of cell adhesion pathways in immune function is interesting in the context of health behaviors. We know that activation of the immune system to target areas of inflammation or infection involves leukocyte recruitment and transmigration to the site of activity via the vasculature [54]. However, when individuals habitually practice healthy behaviors and levels of systemic inflammation are lower, it is possible that genes associated with the transmigration of immune system cells could be downregulated. Yet, in comparing our results to the current literature, we identified no studies that examined a combined lifestyle intervention among healthy adults with DNA methylation pathway analysis. However, a recent study by Turner et al. examined the impact of chronic versus acute resistance exercise on DNA methylation pathways in the skeletal muscle [55]. Even though this study only used the KEGG pathway database, the results identified several significant pathways related to cancer including those related to proteoglycans, transcriptional regulation, colorectal cancer, and small cell lung cancer. Similarly, in the IPA pathway analysis, we identified pathways related to carcinogenesis (e.g., PI3K/AKT signaling, Wnt/β-catenin, sonic hedgehog, and p53 pathways) and some specific to cancers like bladder, ovarian, and brain. Additional studies are needed to refine understanding of the impact of exercise type, intensity, and duration on molecular mechanisms of immune system cells in the blood.

Overall, our study has several strengths, but also limitations. This study is the first randomized controlled trial to examine the impact of a joint physical activity and dietary intervention on DNA methylation using the Illumina 850 k EPIC chip over three time points. The limitations of this study include a relatively small sample size, which likely curtails our ability to detect small changes in methylation status. This study also included both White and African-American participants, but the sample size was not large enough to allow for stratification. Furthermore, there is always a chance for inaccurate self-report of dietary habits. Future studies will include a larger sample and additional measurement time points during the intervention as well as following its conclusion. This will allow us to examine the persistence of the observed changes in DNA methylation.

## Conclusions

The results demonstrate that the MBC2 intervention, which improved diet and physical activity among inactive adults with suboptimal diet quality, impacts patterns of DNA methylation in gene regions related to inflammation, carcinogenesis, and chronic disease. Understanding the biological mechanisms affected by lifestyle interventions may help us to better identify individuals who may most benefit, on a molecular level, from healthy lifestyle change. These preliminary results will inform future studies to examine DNA methylation as a potential biomarker identifying populations that may particularly benefit from incorporating health behavior change into plans for precision prevention of chronic diseases.

## Methods

### Make Better Choices 2 (MBC2) study population and intervention design

MBC2 was a 9-month randomized controlled trial (RCT) conducted between 2012 and 2014 among adults (*n* = 68) aged 18–65 with non-optimal levels of all of 4 lifestyle behaviors: fruits/vegetables, saturated fats, sedentary leisure screen time, and moderate-vigorous physical activity (MVPA) [40, 56]. Specifically, this included the following thresholds for diet: < 5 servings of fruits and vegetables per day and ≥ 8% daily calories from saturated fat; physical activity: < 150 min per week MVPA; and sedentary behavior: > 120 min per week of leisure screen time from sources such as television or video games [40]. The MBC2 participants were randomly assigned to one of three 12-week treatments using a smartphone application and remote coaching to (1) increase MVPA while improving fruits/vegetables and sedentary leisure (simultaneous), (2) improve fruits/vegetables and sedentary leisure first, followed by MVPA (sequential), or (3) improve stress and sleep (control). The study examined a standardized composite diet and physical activity improvement score measured by 1-week assessments when participants wore an accelerometer and self-monitored all behaviors on a smart phone. The primary study outcome was sustained improvement from baseline through 9-month follow-up, and the secondary outcomes were improvement in individual diet and activity behaviors. In the original study, sequential and simultaneous interventions produced similar and sustained diet and activity improvement compared to control (*p* < 0.001), and the effects were comparable between intervention groups [40].

### DNA methylation

We examined patterns of DNA methylation in whole blood samples from 68 participants who had the most complete repeated assessments of behavior and blood, and, who for intervention participants, showed the most behavior change on composite MBC2 outcome measurement. These participants included 25 from the simultaneous intervention arm, 31 from sequential intervention arm, and 12 from the control group. We conducted DNA methylation profiling at three time points (baseline, 3 months, and 9 months) for each participant. We analyzed 204 blood samples for this study. We extracted DNA from the whole blood using the QIAamp DNA Blood Kit (QIAGEN, Valencia, CA, USA). Bisulfite conversion was completed using the EZ-96 DNA Methylation Kit (Zymo Research, Orange, CA, USA). We then measured DNA methylation of CpG probes using Illumina EPIC BeadChip (850 k array) [57].

We collaborated with the University of Chicago Center for Research Informatics for data processing, quality control, and statistical analysis. The bioinformatics and data analysis workflow are shown in Additional file 1: Figure S1. First, we imported the raw methylation data from IDAT files using *read.metharray.exp* function implemented in *minfi* package. For quality control, we calculated the detection *p* value for every CpG in every sample and plotted the mean detection *p* value for each sample, which allowed us to assess the general quality of the samples in terms of the overall signal reliability. We found no low-quality samples in this study. For normalization, we first performed the background correction using *Noob* method from *minfi* package [58, 59]. Since we are comparing different blood samples, which are globally relatively similar, we applied the *preprocessQuantile* method to our data. This function implements a stratified quantile-normalization procedure, which we applied to the methylated and unmethylated signal intensities separately to account for different probe types. We obtained the normalized data in two forms, beta-value (the ratio of the methylated probe intensity and the sum of methylated and unmethylated probe intensities), and M-value (log2 ratio of the intensities of methylated probe versus unmethylated probe).

We also filtered poor-performing probes before differential methylation analysis. We removed probes based on the following criteria: (1) detection *p* value < 0.01, (2) 5% samples with a beadcount < 3, (3) no guanine or cytosine bases, (4) probes on the sex chromosomes, (5) probes with SNPs at CpG site or single-base extension site, (6) polymorphic probes [60], (7) cross-reactive probes [60, 61], and (8) probes that align to multiple locations [62]. Following this filtering, we reduced the number of probes from 866,836 to 749,545 for downstream analysis. Moreover, we examined batch effects using singular-value decomposition (SVD) and multi-dimensional scaling plot. To account for potential batch effects consisting of both categorical and continuous covariates, we applied the surrogate variable analysis (SVA) method implemented in *sva* package to adjust the normalized M-values for any known, unknown, unmodeled, or latent sources of noise, including demographic and other participant characteristics [63].

### Statistical analysis

We conducted region-based differential methylation analysis. While examination of probe-wise differential methylation has utility when single-CpG sites are associated with a phenotype, differentially methylated regions (DMRs) are often more predictive. This is because small differences at any individual site are persistent across a region, statistical power to detect them may be greater in regional analysis compared to probe-wise analysis. Thus, we conducted differential methylation regional analysis for 11 comparisons using the *DMRcate* package [64]. The differentially methylated regions were identified using an FDR adjusted *p* value of 0.05 as well as Stouffer transformed values. We compared control versus pooled sequential and simultaneous intervention groups at baseline, 3 months, and 9 months following the intervention period. We also compared the sequential versus simultaneous groups at 3- and 9-month visits.

For pathway analysis, we then converted the regions to annotated genomic ranges, which uses the genome annotations to annotate overlapping promoter regions (± 2000 bp from TSS). We annotated regions with Ensembl Genome Release 75 (February 2014). To gain an understanding of the biological processes that the differentially methylated CpGs may be involved in, we performed gene ontology, KEGG pathway, and MSigDB canonical pathway (http://software.broadinstitute.org/gsea/msigdb) analyses using the *gometh* function in the *missMethyl* package. We also performed Ingenuity Pathway Analysis (IPA) core analysis. We conducted all analyses using R version 3.4.1 and Bioconductor version 3.5.

## Additional file

Additional file 1: **Figure S1.** Bioinformatics and data analysis pipeline. **Table S1.** Top 40 differentially regions between control vs. pooled sequential/simultaneous at 3 months. **Table S2.** Top 40 differentially regions between control vs. pooled sequential/simultaneous at 9 months. (DOCX 710 kb)

## Data Availability

The datasets used during the current study are available from the corresponding author on reasonable request.
