# Supplementary material for: Impact of a diet and activity health promotion intervention on regional patterns of DNA methylation
Source: Clin Epigenetics. 2019 Sep 11;11:133. doi: 10.1186/s13148-019-0707-0 (PMC6737702; doi:10.1186/s13148-019-0707-0)
Supplement: Supplementary file 1 — Figure S1. Bioinformatics and data analysis pipeline. Table S1. Top 40 differentially regions between control vs. pooled sequential/simultaneous at 3 months. Table S2. Top 40 differentially regions between control vs. pooled sequential/simultaneous at 9 months. (DOCX 710 kb) [file 13148_2019_707_MOESM1_ESM.docx]

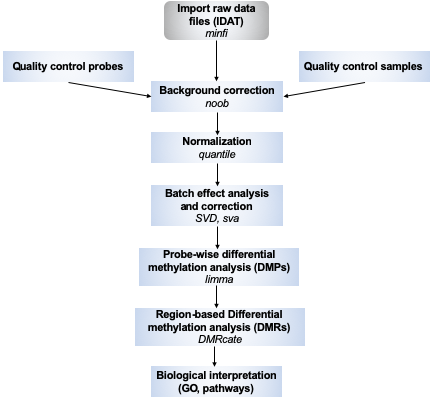


Figure S1. Bioinformatics and data analysis pipeline

Table S1. Top 40 differentially regions between control vs. pooled sequential/simultaneous at three-months

| **Chromosome and coordinates** | **# CpGs** | **FDR** | **Stouffer** | | **β (max)** | **β (mean)** |
| --- | --- | --- | --- | --- | --- | --- |
| chr1:228756711-228756714 | 2 | 0.0036 | | 0.7466 | -0.1183 | -0.1183 |
| chr19:37463254-37463280 | 2 | 0.0012 | | 0.7670 | 0.0787 | 0.0549 |
| chr20:58662458-58662959 | 2 | 0.0029 | | 0.7729 | -0.0878 | -0.0791 |
| chr3:119499749-119500059 | 2 | 0.0005 | | 0.8361 | 0.0982 | 0.0625 |
| chr1:229764652-229764706 | 2 | 0.0059 | | 0.8873 | 0.0622 | 0.0351 |
| chr2:105990524-105991411 | 3 | 0.0013 | | 0.8891 | 0.0539 | 0.0448 |
| chr5:488398-488527 | 2 | 0.0141 | | 0.8900 | 0.0660 | 0.0493 |
| chr3:46599958-46600244 | 2 | 0.0147 | | 0.8921 | 0.0738 | 0.0568 |
| chr1:187445635-187445942 | 2 | 0.0233 | | 0.8955 | -0.0617 | -0.0354 |
| chr4:185369135-185370076 | 3 | 0.0029 | | 0.8977 | -0.1266 | -0.1005 |
| chr7:64126099-64126140 | 2 | 0.0237 | | 0.9007 | -0.0848 | -0.0552 |
| chr5:36662804-36662950 | 3 | 0.0002 | | 0.9040 | -0.0568 | -0.0455 |
| chr22:49875144-49875295 | 2 | 0.0286 | | 0.9242 | -0.0641 | -0.0498 |
| chr1:31256028-31256250 | 2 | 0.0302 | | 0.9259 | 0.1417 | 0.1031 |
| chr6:1684515-1684567 | 2 | 0.0335 | | 0.9317 | 0.0306 | 0.0192 |
| chr2:37232322-37232596 | 2 | 0.0473 | | 0.9462 | -0.1293 | -0.0970 |
| chr16:87682036-87682142 | 2 | 0.0203 | | 0.9620 | -0.0970 | -0.0922 |
| chr8:145164473-145164991 | 2 | 0.0311 | | 0.9724 | 0.0867 | 0.0801 |
| chr8:51306070-51306693 | 3 | <0.0001 | | 0.9855 | 0.2099 | 0.0870 |
| chr16:12660858-12661268 | 2 | 0.0014 | | 0.9975 | 0.0126 | 0.0060 |
| chr17:149484-149488 | 2 | 0.0014 | | 0.9975 | 0.0528 | 0.0311 |
| chr11:379296-379914 | 3 | 0.0106 | | 0.9985 | 0.0515 | 0.0450 |
| chr3:14731751-14731951 | 2 | 0.0182 | | 0.9994 | 0.0755 | 0.0384 |
| chr5:134844273-134844306 | 2 | 0.0224 | | 0.9995 | -0.0517 | -0.0283 |
| chr1:209826863-209827055 | 2 | 0.0075 | | 0.9997 | -0.0441 | -0.0242 |
| chr1:245524538-245524769 | 2 | 0.0048 | | 0.9997 | 0.0231 | 0.0209 |
| chr15:33534869-33534982 | 2 | 0.0231 | | 0.9997 | -0.0729 | -0.0468 |
| chr4:175241684-175241753 | 2 | 0.0041 | | 0.9997 | -0.1390 | -0.0878 |
| chr7:51084006-51084330 | 2 | 0.0117 | | 0.9997 | 0.0410 | 0.0244 |
| chr3:69248890-69249276 | 2 | 0.0025 | | 0.9998 | -0.0767 | -0.0619 |
| chr12:3259783-3260174 | 2 | 0.0056 | | 0.9999 | -0.0443 | -0.0389 |
| chr12:117349196-117349264 | 2 | 0.0386 | | 0.9999 | -0.0059 | -0.0036 |
| chr5:140810726-140810805 | 2 | 0.0422 | | 0.9999 | -0.0541 | -0.0498 |
| chr1:160141079-160141163 | 2 | 0.0452 | | 0.9999 | 0.0157 | 0.0135 |
| chr5:769812-770095 | 2 | 0.0308 | | 0.9999 | 0.0281 | 0.0129 |
| chr2:11817098-11817180 | 2 | 0.0240 | | 0.9999 | -0.0583 | -0.0380 |
| chr20:60240263-60240403 | 2 | 0.0371 | | 0.9999 | -0.0284 | -0.0274 |
| chr17:47296912-47296970 | 2 | 0.0302 | | 0.9999 | -0.0615 | -0.0414 |
| chr9:140175219-140175393 | 2 | 0.0202 | | 0.9999 | -0.0275 | -0.0188 |

Table S2. Top 40 differentially regions between control vs. pooled sequential/simultaneous at nine-months

| **Chromosome and coordinates** | | **# CpGs** | **FDR** | | **Stouffer** | **β (max)** | | | **β (mean)** |
| --- | --- | --- | --- | --- | --- | --- | --- | --- | --- |
| chr8:51306070-51306693 | 3 | | <0.0001 | 0.0904 | | | 0.2066 | 0.1012 | |
| chr3:150265617-150265620 | 2 | | <0.0001 | 0.1754 | | | 0.0351 | 0.0327 | |
| chr3:44636619-44636844 | 2 | | 0.0008 | 0.3556 | | | 0.0465 | 0.0267 | |
| chr22:49875144-49875295 | 2 | | 0.0040 | 0.3980 | | | -0.0775 | -0.0574 | |
| chr16:1477870-1478718 | 3 | | 0.0001 | 0.4290 | | | -0.0667 | -0.0434 | |
| chr19:7813422-7813963 | 3 | | <0.0001 | 0.4426 | | | -0.0800 | -0.0468 | |
| chr13:101173617-101174420 | 8 | | <0.0001 | 0.4572 | | | -0.0929 | -0.0537 | |
| chr11:1903176-1903333 | 2 | | 0.0047 | 0.4611 | | | -0.0586 | -0.0486 | |
| chr4:185369135-185370076 | 3 | | 0.0030 | 0.4752 | | | -0.1271 | -0.1000 | |
| chr4:175241684-175241753 | 2 | | 0.0071 | 0.4980 | | | -0.1368 | -0.0847 | |
| chr17:72915897-72916509 | 3 | | 0.0009 | 0.5005 | | | -0.0636 | -0.0595 | |
| chr1:228756711-228756714 | 2 | | 0.0141 | 0.5057 | | | -0.1112 | -0.1106 | |
| chr12:133303923-133304061 | 2 | | 0.0084 | 0.5096 | | | -0.0121 | -0.0096 | |
| chr20:58662458-58662959 | 2 | | 0.0150 | 0.5168 | | | -0.0930 | -0.0740 | |
| chr5:36662804-36662950 | 3 | | 0.0006 | 0.5209 | | | -0.0580 | -0.0446 | |
| chr19:37463254-37463280 | 2 | | 0.0062 | 0.5271 | | | 0.0741 | 0.0505 | |
| chr14:34162098-34162679 | 2 | | 0.0001 | 0.5281 | | | 0.0231 | 0.0127 | |
| chr11:66027798-66028483 | 2 | | 0.0217 | 0.5281 | | | 0.0201 | 0.0181 | |
| chr3:57533508-57533749 | 2 | | 0.0013 | 0.5289 | | | 0.0265 | 0.0178 | |
| chr7:158250911-158250978 | 2 | | 0.0295 | 0.5333 | | | 0.1491 | 0.1204 | |
| chr12:124873347-124874007 | 5 | | <0.0001 | 0.5350 | | | -0.1335 | -0.0635 | |
| chr11:2212133-2212225 | 2 | | 0.0260 | 0.5473 | | | -0.0789 | -0.0773 | |
| chr4:940614-941054 | 3 | | 0.0004 | 0.5578 | | | -0.1164 | -0.0756 | |
| chr12:78573360-78573389 | 2 | | 0.0154 | 0.5579 | | | 0.0541 | 0.0499 | |
| chr2:90138273-90138295 | 2 | | 0.0054 | 0.5622 | | | 0.0102 | 0.0084 | |
| chr1:113045271-113045452 | 2 | | 0.0079 | 0.5643 | | | -0.0430 | -0.0359 | |
| chr20:43859414-43859502 | 3 | | 0.0004 | 0.5711 | | | -0.0428 | -0.0301 | |
| chr6:42946146-42946178 | 2 | | 0.0025 | 0.5730 | | | -0.0401 | -0.0318 | |
| chr3:46599958-46600714 | 3 | | 0.0021 | 0.5746 | | | 0.0731 | 0.0404 | |
| chr6:93433536-93433628 | 2 | | 0.0142 | 0.5790 | | | 0.0387 | 0.0336 | |
| chr4:188963260-188963324 | 2 | | 0.0222 | 0.5797 | | | -0.0778 | -0.0539 | |
| chr11:379296-379455 | 2 | | 0.0054 | 0.5815 | | | 0.0516 | 0.0442 | |
| chr8:37555391-37555577 | 2 | | 0.0079 | 0.5833 | | | -0.0390 | -0.0314 | |
| chr8:37082441-37082537 | 2 | | 0.0227 | 0.5836 | | | -0.0674 | -0.0611 | |
| chr10:132099553-132100019 | 2 | | 0.0194 | 0.5962 | | | 0.1338 | 0.1284 | |
| chr2:17718449-17718971 | 2 | | 0.0194 | 0.5968 | | | 0.0466 | 0.0166 | |
| chr17:30873196-30873447 | 2 | | 0.0336 | 0.6066 | | | -0.0531 | -0.0488 | |
| chr15:26341034-26341252 | 2 | | 0.0140 | 0.6108 | | | -0.0471 | -0.0406 | |
| chr9:20624418-20624421 | 2 | | 0.0154 | 0.6124 | | | -0.0649 | -0.0536 | |
